# Using a three-compartment model improves the estimation of iohexol clearance to assess glomerular filtration rate

**DOI:** 10.1038/s41598-018-35989-x

**Published:** 2018-12-07

**Authors:** Max Taubert, Natalie Ebert, Peter Martus, Markus van der Giet, Uwe Fuhr, Elke Schaeffner

**Affiliations:** 1 Department I of Pharmacology, University Hospital Cologne, Cologne, Germany; 2 Institute of Public Health, Charité – Universitätsmedizin Berlin, corporate member of Freie Universität Berlin, Humboldt-Universität zu Berlin, and Berlin Institute of Health, Berlin, Germany; 3 Institute for Clinical Epidemiology and Applied Biostatistics, University Hospital Tübingen, Tübingen, Germany; 4 Department of Nephrology, Charité – Universitätsmedizin Berlin, corporate member of Freie Universität Berlin, Humboldt-Universität zu Berlin, and Berlin Institute of Health, Berlin, Germany

## Abstract

Plasma clearance of iohexol is a key tool to precisely determine glomerular filtration rate (GFR) in clinical research and clinical practice. Despite evidence that iohexol pharmacokinetics are described best by three-compartment models, two-compartment approaches (Schwartz approach) are customary, which might result in avoidable bias and imprecision. We aimed to provide a population pharmacokinetic (popPK) model of iohexol by re-evaluating data from the Berlin Initiative Study (BIS) to compare respective clearance estimates to the Schwartz approach and to assess the impact of revised clearance estimates on the BIS equations. A popPK model was developed based on iohexol plasma samples (8–10 per subject, iohexol dose 3235 mg) from 570 elderly patients. A three-compartment model appropriately described the pharmacokinetics of iohexol (clearance 57.4 mL/min, CV 33%). Compared to the three-compartment model, clearance values were overestimated by the Schwartz approach (bias 6.5 mL/min), resulting in limited effects on regression coefficients of the BIS equations (e.g., proportionality factor of BIS2 changed from 767 to 720). Predictions based on the BIS2 equation were biased (5.4 mL/min/1.73 m²) and the sensitivity to detect a GFR < 60 mL/min/1.73 m² was low compared to the revised equation (72% versus 89%). Three-compartment models should be employed to assess iohexol pharmacokinetics.

## Introduction

Because of its favorable pharmacokinetic properties and better availability compared to inulin, plasma clearance of iohexol has become a key tool to determine glomerular filtration rate (GFR)^1^ accurately. Recently published GFR prediction equations, such as the Berlin Initiative Study (BIS)^2^, rely on iohexol clearance estimates obtained via the Schwartz approach^3^. This approach assumes a double-exponential decay of iohexol plasma concentrations and thus represents a two-compartment model. However, there is evidence that iohexol pharmacokinetics is best described by a three-compartment model^4^, suggesting that estimates obtained by the Schwartz approach are subject to avoidable bias. Specifically, neglecting a third compartment with quick equilibration to plasma might result in over-estimation of GFR values and, ultimately, biased GFR prediction equations. The widespread use of the Schwartz approach might partially result from its simplicity and modest requirements regarding the number and timing of plasma samples to be taken, while extensive sampling is needed to fit three-compartment models. However, population pharmacokinetic methods reduce the requirements on sampling designs by applying mixed-effects modeling approaches and have become the gold standard in pharmacokinetic evaluations. The objective of the present evaluation was to assess the impact of neglecting a third compartment on iohexol clearance estimates and GFR prediction equations, based on data from the previously conducted BIS study^5^.

## Results

To assess the need for more complex models of iohexol pharmacokinetics, the evaluation was carried out in three main steps. First, population pharmacokinetic models were evaluated based on iohexol plasma concentration measurements. Second, iohexol clearance estimates obtained from the standard two-compartment Schwartz approach were compared to two- and three-compartment population pharmacokinetic clearance estimates. Third, the impact of changes in clearance estimates on the BIS1 and BIS2 equations was assessed.

### Population pharmacokinetic two- and three-compartment models

Based on visual inspection of the data, seven of 570 subjects with implausible concentration-time curves were excluded from the analysis. For these subjects, concentrations were either steadily increasing over several hours, indicating para-vascular administration, or initial concentrations were very high and decreased rapidly (e.g. corresponding to an implausible elimination half-life of only 20 minutes).

**Figure 1:**
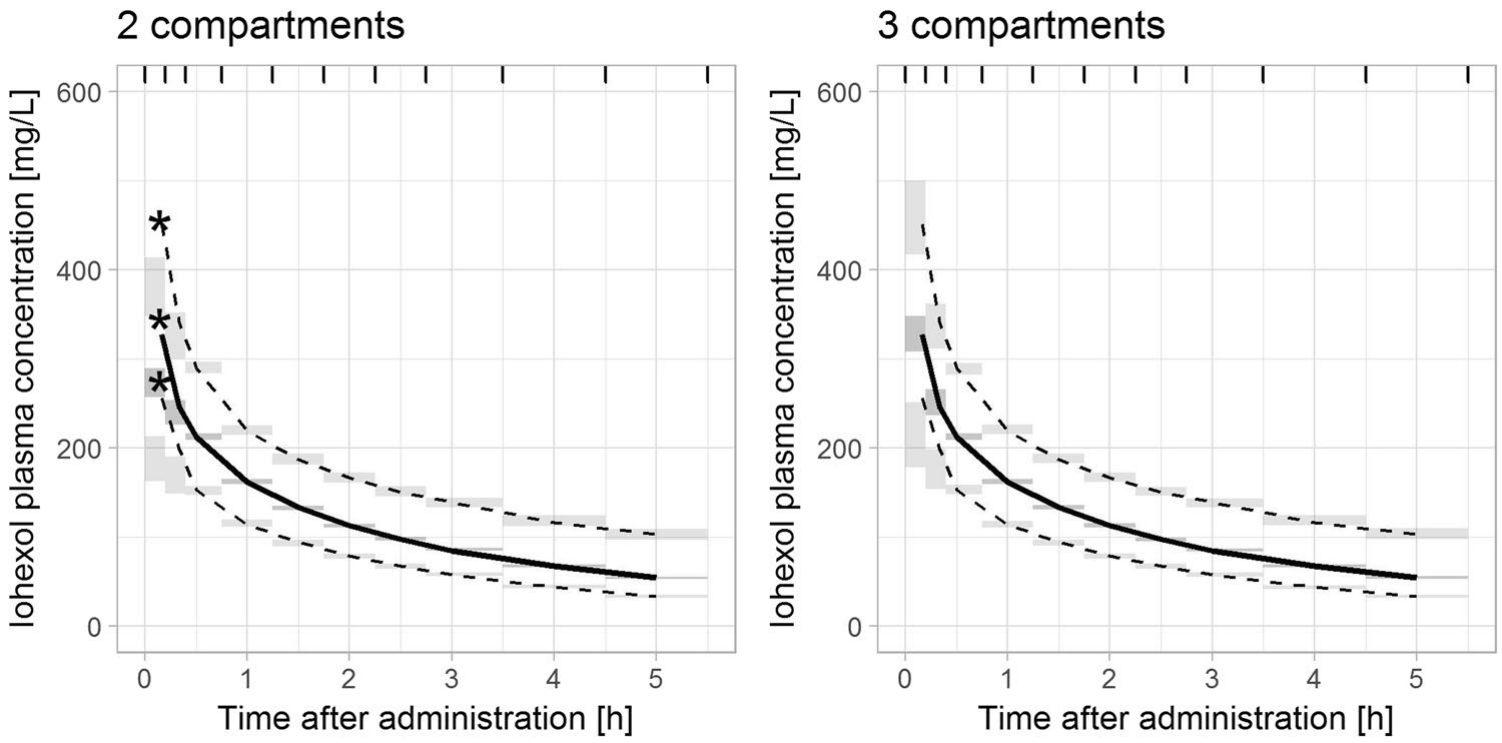
Visual predictive checks of the two- and three-compartment population pharmacokinetic models. Visual predictive checks of the two-compartment (left side) and three-compartment (right side) model. Solid (dashed) lines represent medians (5%, 95% percentiles) of observed concentrations; grey areas represent 95% confidence intervals of 5%, 50% and 95% percentiles predicted by the model. For a correctly specified compartmental model, observed medians should lie inside the middle grey boxes. Observed 95% percentiles should lie within the upper and 5% percentiles within the lower grey boxes. For the two-compartment model, model-predictions of early measurements are relevantly lower than the observed data, which is indicated by an asterisk. Thus, the two-compartment model is clearly misspecified with respect to early concentration measurements. For the three-compartment model, only early low concentrations are not predicted well, indicating that misspecifications mostly disappeared.

**Figure 2:**
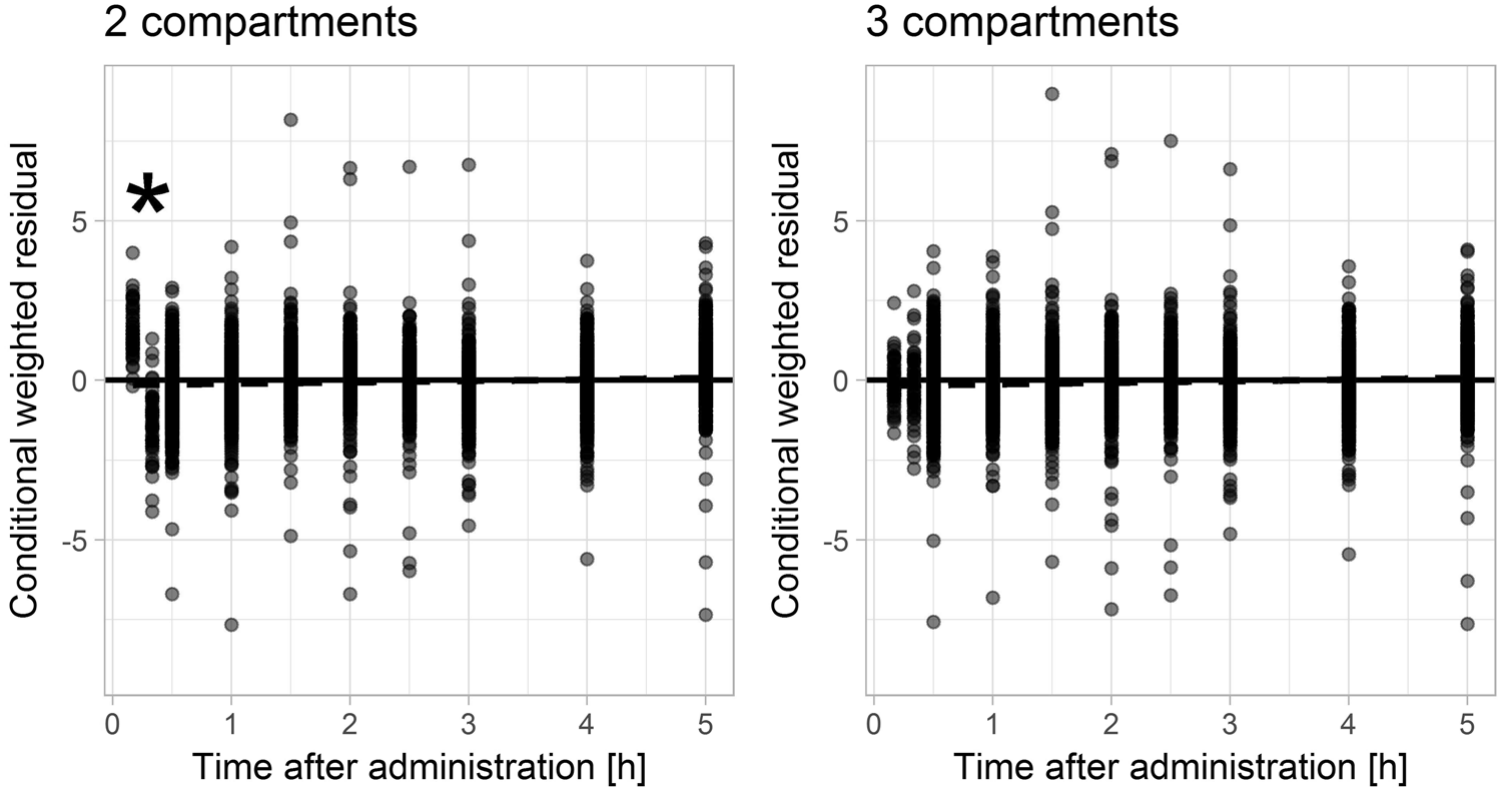
Residual plots indicating a misspecification of the two-compartment population pharmacokinetic model compared to the three compartment model. Conditional weighted residuals versus time after administration of iohexol from the two-compartment (left side) and three-compartment (right side) model. A clear misspecification is apparent for early concentrations in the two-, but not in the three-compartment model (indicated by asterisk).

**Figure 3:**
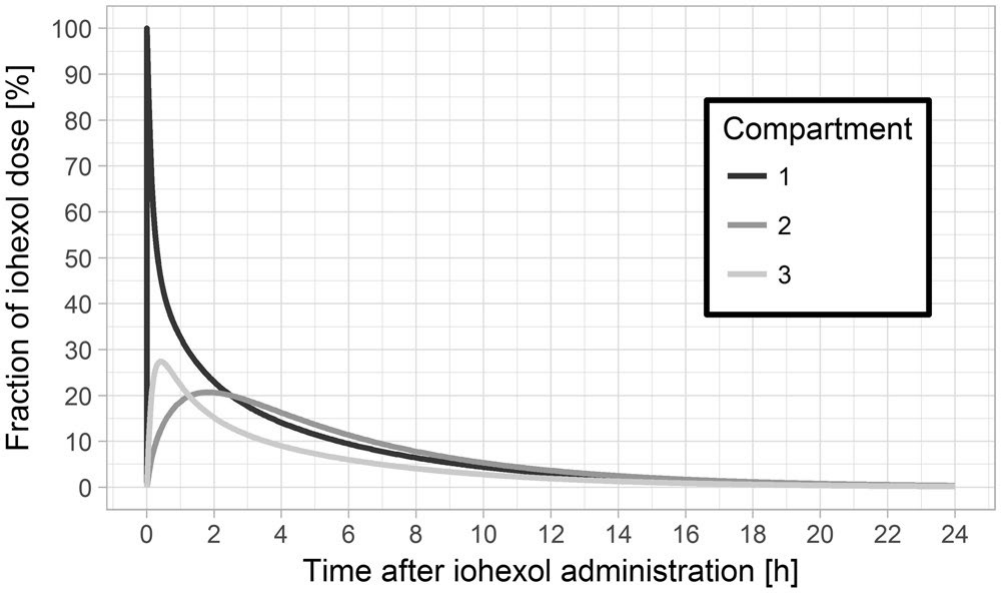
Amounts of iohexol in the three compartments over time. Median percentage of iohexol amounts (relative to the administered dose) in the compartments 1, 2 and 3 over time as predicted by the three-compartment population pharmacokinetic model. The amount of iohexol in the third compartment increases quickly, thus yielding a fast, initial decrease of plasma concentrations.

The population pharmacokinetic evaluation revealed a clear misspecification when using a model with two compartments only. Specifically, visual predictive checks indicated that the model-predicted initial concentrations were significantly lower than observed iohexol concentrations when assuming two compartments (Fig. 1, left side). This was further confirmed by weighted residuals, which showed that measurements after 10 and 20 minutes were under- and overpredicted, respectively (Fig. 2, left side). By adding a third compartment, misspecifications nearly completely disappeared (Figs 1 and 2, right side) and the model improved significantly in terms of objective function value (decrease by 617 points). Further increases in model complexity, e.g. non-linear elimination or further compartments, were not supported by the data. The final model comprised three compartments, first-order elimination, a combined error model and a full variance-covariance matrix of random effects. As illustrated in Fig. 3, the third compartment mainly accounted for a steeper initial concentration decay. To assess whether the distinct differences between two- and three-compartment models mainly resulted from early measurements, the model evaluation was repeated without samples taken earlier than 30 minutes after iohexol infusion. Although the previously observed misspecifications in VPCs and residual plots vanished upon removal of early measurements, parameter point estimates related to clearance changed by less than 1%. Therefore, model parameter estimates were not relevantly affected by the in- or exclusion of early measurements and bias of the resulting estimates did not increase.

Based on the final three-compartment model, the median population value of iohexol clearance was 57.4 (55.6–59.1) mL/min with a coefficient of variation (CV) of 33 (31–36) % (medians and 95% confidence intervals from bootstrap). Individual clearance estimates (empirical Bayes estimates, EBEs) ranged from 15 to 136 mL/min, median 61 mL/min, with an eta shrinkage of 2% and a median (95% CI) relative standard error of 1.3 (0.6–4.9) %. Equilibration to the third compartment, as described by the inter-compartmental clearance (Q) of 12.0 (9.1–14.4) L/h and peripheral volume (V) of 3.3 (2.7–3.8) L, was fast compared to the second compartment (Q of 3.5 [3.0–3.8] L/h, V of 5.4 [5.2–5.6] L). For other model parameters, see Supp. Table 1.

### Comparison of Schwartz and population pharmacokinetic estimates

In the next step, clearance estimates obtained from the Schwartz approach were compared to estimates from the two- and three-compartment models to elucidate whether the identified model misspecifications translated into relevant differences in clearance estimates.

**Figure 4:**
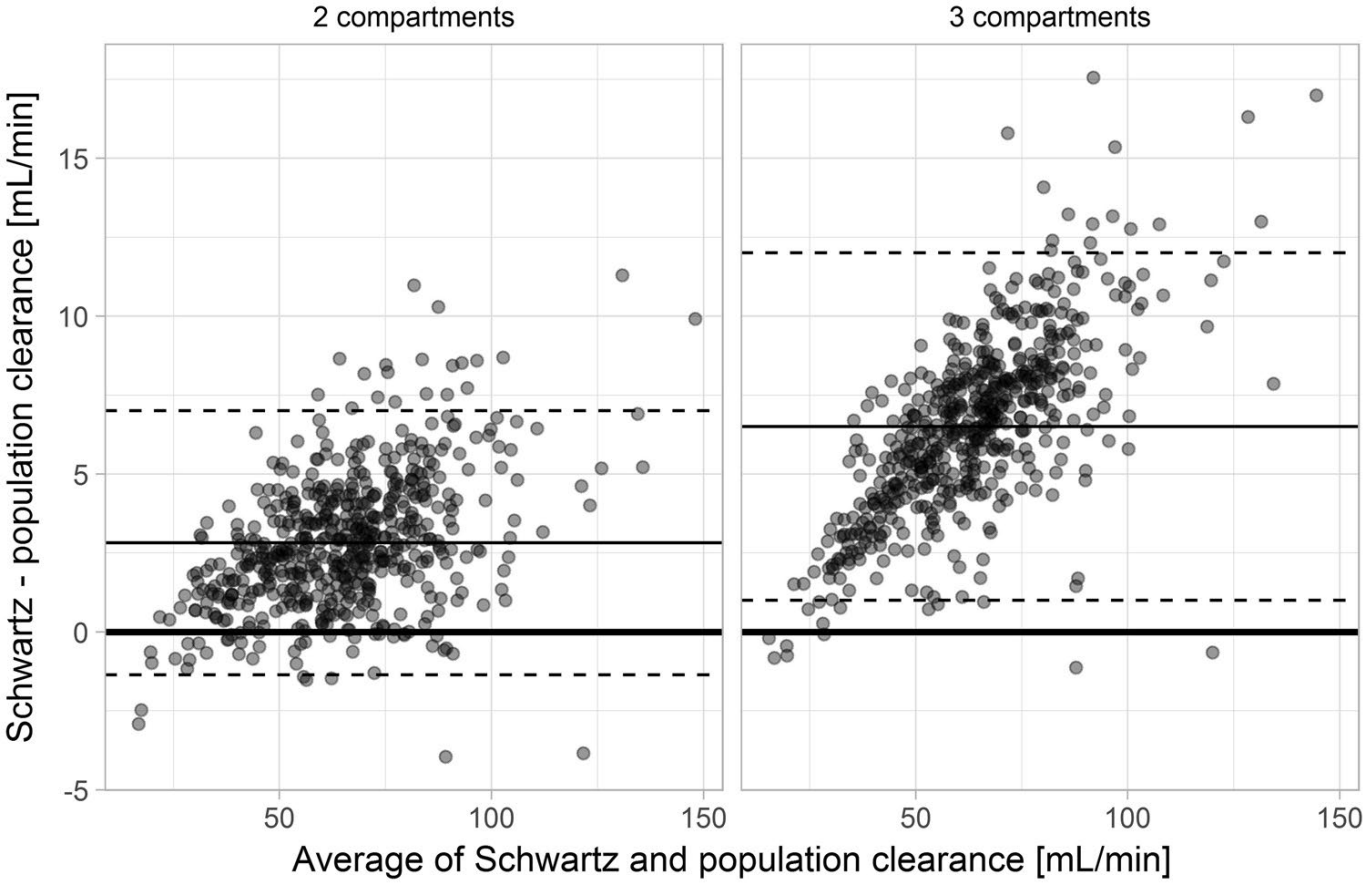
Bland-Altman plots of new and revised clearance estimates. Bland-Altman plot of clearance estimates obtained from the Schwartz approach compared to clearance estimates from a population pharmacokinetic two-compartment (left side) and three-compartment (right side) model. The bias (2.8 ml/min for the 2 compartment and 6.5 ml/min for the 3-compartment model) is represented by the thin solid line, the upper and lower limits of agreement by the dashed lines.

Compared to the three-compartment population pharmacokinetic model, the Schwartz approach overestimated clearances with a bias of 6.5 mL/min, root mean squared error (RMSE) of 7.1 mL/min and mean absolute error (MAE) of 6.5 mL/min. Overpredictions increased with increasing GFR. For example, the bias for subjects with a clearance ≥60 mL/min reached 8.0 mL/min. Similar but quantitatively smaller overpredictions were also observed when comparing Schwartz estimates to the two-compartment population pharmacokinetic model (Fig. 4).

### Influence of revised iohexol clearance estimates on the BIS equations

In the third step, models resembling the BIS2 and BIS1 equations with serum creatinine, age, gender, and with and without cystatin C were evaluated with regard to their aptness to predict iohexol clearance estimates. The following results refer to models fitted to iohexol clearance estimates from the Schwartz or population pharmacokinetic two- or three-compartment models while model (cross-)validation was performed on clearance values from the three-compartment model. Bias was highest for equations based on Schwartz estimates while equations based on the three-compartment model were nearly unbiased (Table 1). Consistently, RMSE and MAE were lowest for equations based on the three-compartment model.

**Table 1:** Performance of BIS1 and BIS2 equations to predict iohexol clearance estimates obtained by different methods.

Method	Equation	Bias [mL/min/1.73 m²]	RMSE [mL/min/1.73 m²]	MAE [mL/min/1.73 m²]
Schwartz	BIS1	5.3	10.0	7.9
	BIS2	5.4	9.0	7.2
Pop PK, 2 compartments	BIS1	2.7	8.8	6.7
	BIS2	2.9	7.7	5.9
Pop PK, 3 compartments	BIS1	−0.6	8.3	6.1
	BIS2	−0.4	7.1	5.2

Bias, root mean squared error (RMSE) and mean absolute error (MAE) of the BIS1 and BIS2 equations fitted to clearance estimates obtained from the standard Schwartz approach and population pharmacokinetic (Pop PK) approaches with two and three compartments. Bias, RMSE and MAE were calculated using cross-validation based on individual clearance estimates obtained from the three-compartment model.

Changes in regression coefficients were highest for *β*_0_, i.e. the intercepts on the log-scale (proportionality factors on the original scale), while coefficients related to creatinine, cystatin C, age and gender changed only marginally (Table 2). Despite the limited effect on regression coefficients, the sensitivity to detect a GFR below 60 mL/min/1.73 m² increased from 72% based on Schwartz estimates to 89% based on the revised clearance estimates from the three-compartment model (equation with cystatin C). The sensitivity to detect a GFR below 30 mL/min/1.73 m² increased from 50% to 65%.

**Table 2:** Comparison of regression coefficients.

	*β*_0_ (constant)	*β*_1_ (creatinine)	*β*_2_ (cystatin C)	*β*_3_ (age)	gender
BIS1 old	3736	−0.87	—	−0.95	82%
BIS1 new	3232	−0.85	—	−0.94	82%
BIS2 old	767	−0.40	−0.61	−0.57	87%
BIS2 new	720	−0.40	−0.59	−0.58	87%

Published (old) and re-estimated (new) regression coefficients of BIS equations with (BIS2) and without (BIS1) cystatin C of the following form: $GFR\,[mL/min/1.73{m}^{2}]=$ ${\beta }_{0}\,\ast \,c{r}^{{\beta }_{1}}\,\ast \,c{c}^{{\beta }_{2}}\,\ast \,ag{e}^{{\beta }_{3}}\,\ast \,gender(if\,female)$, where cr = creatinine serum concentration, cc = cystatin C serum concentration. Differences in *β*_0_ were more distinct for the BIS1 than for the BIS2 equation.

### Simulations based on the three-compartment model

**Figure 5:**
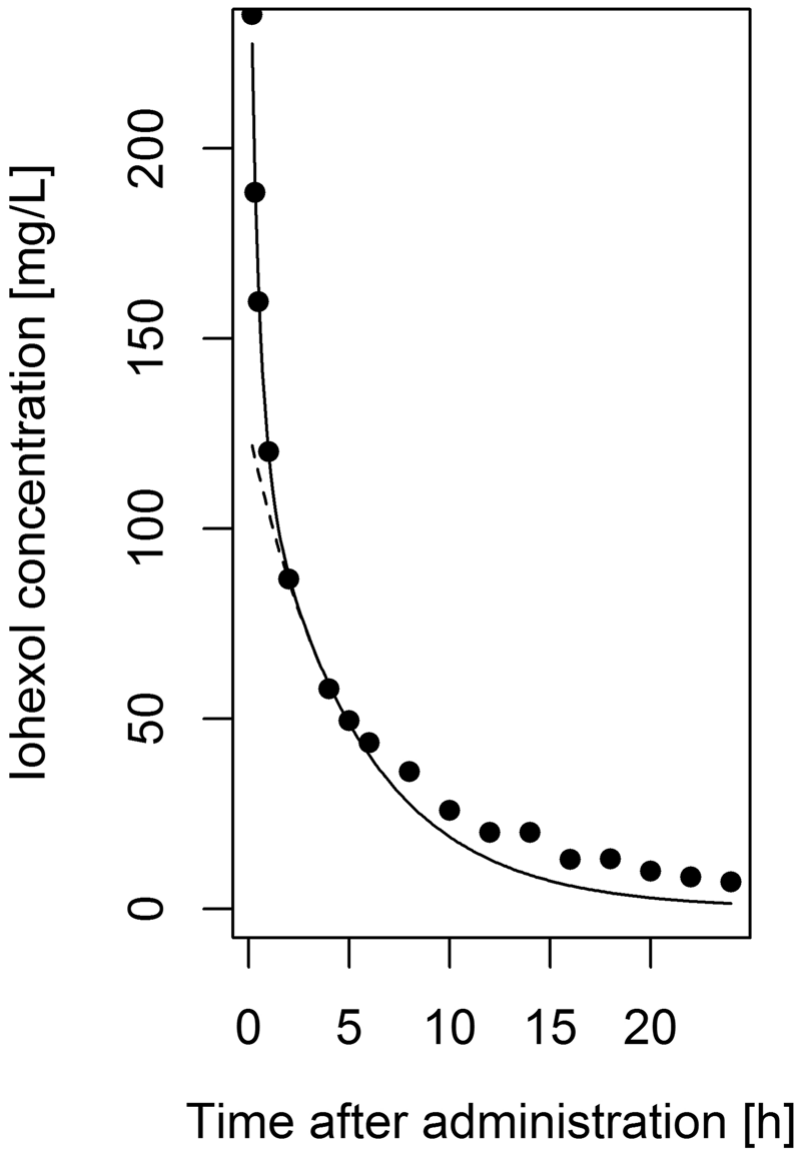
Exemplary plot of the Schwartz approach applied to a simulated three-compartment concentration-time curve. Exemplary plot of simulated concentrations (dots) in a single, virtual subject, and the slow (dashed line) and slow + fast (solid line) components based on the Schwartz approach fitted to concentrations 10, 20, 30, 60, 120, 240 and 300 minutes post-dose. Underprediction is apparent for later concentrations, reflecting the overestimated clearance.

**Figure 6:**
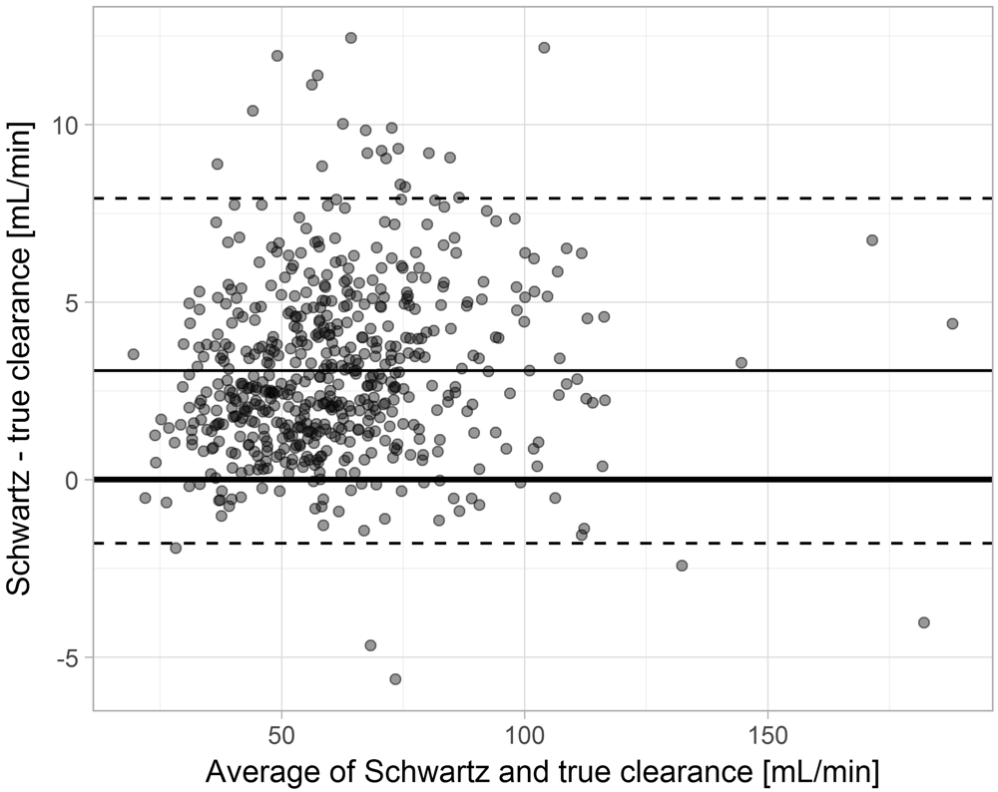
True versus estimated clearances from simulations. Bland-Altman plot of clearance estimates obtained from the Schwartz approach compared to true clearance values from a simulated population pharmacokinetic three-compartment model. The bias (3.1 mL/min) is represented by the solid middle line, the upper and lower limits of agreement by the dashed lines.

Finally, the Schwartz approach was applied to concentration-time profiles that were simulated using the three-compartment model. This simulation enabled to assess whether a perfectly known misspecification could be identified when using the Schwartz approach. By visual inspection, Schwartz’ fits of the simulated iohexol concentration-time profiles were adequate. A typical example is shown in Fig. 5. R squared was high for the slow (0.99 [0.97–1.0]) and fast (0.98 [0.89–1.0]) components in most simulated subjects (median [95% CI]). However, iohexol clearance values based on the Schwartz approach overestimated true clearance values with a bias of 3.2 mL/min, RMSE of 4.0 mL/min and MAE of 3.3 mL/min. Bias increased with increasing clearance (Fig. 6). In other words, a seemingly excellent fit to the data did not imply that the Schwartz approach was not misspecified.

## Discussion

In this analysis, the pharmacokinetics of iohexol was best described by a three-compartment model while a two-compartment population pharmacokinetic model was not capable of appropriately describing early iohexol concentrations. Clearance values were overestimated when assuming two compartments. Three-compartment models should be applied to increase the validity of clearance estimates in iohexol evaluations.

In our evaluation, a model with two compartments was biased for early measurements up to 30 minutes after the administration of iohexol while later iohexol concentrations were described well. If a third compartment with quick equilibration to plasma exists, iohexol concentrations initially decrease faster due to “disappearance” in the third compartment. When fitting a two-compartment model to such concentration-time profiles, the fast component also has to capture the rapid initial decay, which yields overestimated clearances. This does, of course, not represent kidney function, but distribution of iohexol in the body, and is therefore not intended. The exclusion of early concentration-time points obfuscates the misspecifications but still yields biased clearance estimates. Thus, the third compartment apparently affects the whole concentration-time curve rather than only early time points. Evaluating residuals is therefore not sufficient to identify a model misspecification if measurements earlier than 30 minutes post-dose are not available. Indeed, fits of two exponential curves to simulated concentration-time curves from a three-compartment model revealed that it was not feasible to identify misspecifications by visual inspection. The overestimated clearance therefore nearly perfectly corrects for the missing third compartment. Clear misspecifications became only apparent in the population pharmacokinetic evaluation since it enables to assess the aptness of compartmental models via simulation-based visual predictive checks. Without VPCs, more samples would be needed to identify whether a compartmental model describes the pharmacokinetics of iohexol sufficiently well.

Although the numerical influence of the method used to estimate iohexol clearance on regression coefficients was marginal, bias in clearance estimates must be expected to have a major impact on the GFR classification of subjects under certain circumstances. For example, approximately 24% of our patient collective had a GFR in the range 60–70 mL/min/1.73 m², which is close to the classification threshold for a mild to moderate chronic kidney disease stage^6^. Correcting *β*_0_ by about 6% from 767 to 720 while ignoring changes in other regression coefficients implies that misclassification occurs in subjects with a predicted GFR of 60 to 64 mL/min/1.73 m². For the participants of the BIS, this meant that about 10% of the subjects would have been falsely classified to have no CKD (assuming the absence of other kidney damage markers) when using the initial BIS2 equation. This would deteriorate further when using the initial BIS1 equation, for which classification would have been incorrect in more than 20% of the patients. Thus, although the bias of the initial equations at first seems marginal for individual patients, the use of eGFR equations for CKD classification implies a remarkable clinical significance at the population level. This simplified example does not even take into account that coefficients of age and cystatin C also changed. For a typical subject with median age (76.9 years), creatinine (0.91 mg/dL) and cystatin C (1.05 mg/L), the predicted GFR would reduce from 65 to 59 mL/min/1.73 m². This again implies a change in CKD classification. Thus, although in general changes in predicted GFR values were limited, their clinical relevance might be high for subjects with a decreased GFR^7^.

Although population pharmacokinetic evaluations are computationally more burdensome, several benefits over evaluations based separately on individual concentration vs. time profiles are evident. A mixed-effects approach not only reduces the number of samples needed per subject, but it also attenuates the effect of outliers on individual estimates and can handle incomplete iohexol concentration-time profiles^8^. In case of a three-compartment model, at least six parameters need to be estimated from the data. Therefore, approaches to fit a three-compartment model to individual data fail unless samples are drawn very extensively. For example, six to eight samples were available per subject in the presented study, which clearly prevented from estimating three-compartment model parameters subject-wise. It has indeed been shown before that three-compartment models might be needed to describe iohexol pharmacokinetics appropriately. For example, Frennby *et al*.^9^ found that a three-compartment model yielded better fits than a one-compartment model in pigs receiving iohexol intravenously. However, this was based on an extensive sampling scheme with early measurements, which would not be feasible in a clinical setting or as part of further studies on a larger scale. Although characterizing the population distribution of parameters was not the primary aim of this study, it would be a prerequisite for developing GFR prediction equations completely based on a mixed-effects framework. This would obviate the need for empirical Bayes estimates, which are calculated by considering both the fit of the individual concentration-time profile and the likelihood of observing certain pharmacokinetic parameters given the population model. Thus, shrinkage towards the population medians can occur if individual data is not informative, which might affect the estimation of regression coefficients in GFR prediction equations. Although avoiding the use of EBEs might be preferable in general, shrinkage was distinctly low in our study and is therefore not expected to have severe influence on calculations based on EBEs. Furthermore, the use of the nonparametric option in NONMEM relaxes parametric assumptions and might thus provide more appropriate individual clearance estimates. The nonparametric option is a combination of a parametric population model and subsequent nonparametric estimation of individual pharmacokinetic parameters based on EBEs from the parametric model as support points. This approach has been shown to perform well even if the assumption of log-normality is violated heavily, e.g. given tri- or quadromodal or heavy-tailed true distributions^10^. However, we observed a nearly perfect match between the observed individual clearance distribution and the theoretical log-normal distribution. Thus, assuming log-normality seems plausible for further evaluations.

A limitation of this study is the limited sample size especially with respect to early concentration measurements, for which data were only available in 52 subjects. Since the misspecification of models with one or two compartments were most distinct for early measurements, these 52 subjects might have had a more pronounced influence on results than the remaining subjects. However, excluding early time points from the evaluation did not have relevant influence on model parameters. Additionally, renal and non-renal clearance could not be distinguished since no data on urinary clearance were available. Although a systematic influence on clearance estimates must be expected, neglecting non-renal clearance presumably has only mild influence on the presented results. For example, it has been shown that non-renal clearance of iohexol typically does not exceed 5%^1^. As an additional limitation and in contrast to the study by Schwartz *et al*.^3^, iohexol syringes were not weighed. Thus, slight deviations from the intended dose of 3235 mg might have occurred, which might have affected the estimated clearances and volumes of distribution. However, variability in the administered doses of iohexol does not affect the main results of this study since the same dose was assumed for all evaluated approaches. Further validation studies are desirable to confirm the aptness of the presented iohexol model.

## Conclusion

Iohexol clearance should be estimated using three-compartment models to avoid relevant bias. Population pharmacokinetic approaches or extensive sampling facilitate the estimation of related pharmacokinetic parameters. The use of population pharmacokinetic modeling techniques might improve GFR prediction equations based on iohexol gold standard. Furthermore, the presented model can be employed to identify the optimum number and timing of samples needed to estimate individual GFR in elderly subjects appropriately.

## Methods

This evaluation was based on data stemming from the previously conducted Berlin Initiative Study^5^, which was approved by the Ethics Committee of Charité Universitätsmedizin Berlin and carried out in accordance with relevant regulations. All participants gave written informed consent.

Three main evaluations were carried out to assess the need for more complex pharmacokinetic models. In the first step, population pharmacokinetic modeling was employed to assess the effect of a different number of compartments on the capability to describe individual iohexol concentration-time profiles. Then, clearance estimates were obtained from the final population pharmacokinetic model(s) and were compared to standard Schwartz estimates^3^. This was ought to provide insight whether the Schwartz approach was linked to avoidable bias and imprecision. Finally, the BIS equations were re-estimated using the new iohexol clearance estimates to assess the effect of the pharmacokinetic modeling approach on the resulting eGFR equations.

For the first step, a population pharmacokinetic model of iohexol was developed using the first-order conditional estimation algorithm (FOCE) and the nonparametric option with NONMEM 7.4.2, R 3.4.0, PsN 4.7.0^11^ and Pirana 2.9.6. Iohexol plasma concentrations after intravenous injection of 3235 mg iohexol as well as demographic/biometric (age, sex, body weight and height) and laboratory data (Serum creatinine and cystatin C) were available from 570 elderly patients participating in the Berlin Initiative Study. Plasma samples to quantify iohexol were taken 30, 60, 90, 120, 150, 180, 240 and 300 minutes after intravenous injection in all subjects. Additional plasma samples taken 10 and 20 minutes after iohexol infusion were available from 52 patients. In short, the Berlin Initiative Study comprised 2,069 subjects with age ≥70 years randomly sampled from a large German health insurance database^5^. For estimating the BIS equations, a subsample of 610 subjects agreed to have iohexol clearance measured. The final sample of 570 subjects resulted after excluding 40 subjects with incomplete iohexol measurement, insufficient Schwartz fit or implausible mGFR^2^. The dataset has previously been evaluated using the Schwartz approach; please refer to the respective publication for more information^2^. The structural and stochastic model was developed step-wise, starting with one compartment without random effects. Further compartments as well as random inter-individual variability (IIV) were introduced if the respective introduction was linked to a decrease in objective function value (OFV) by at least 6.63 points, stable parameter estimates and improvements in visual predictive checks (VPC). VPCs are an established technique to assess whether a population pharmacokinetic model appropriately predicts pharmacokinetic data, which enables to assess whether structural (e.g., the number of compartments) and distributional assumptions are reasonable. In short, theoretical concentration-time profiles are simulated from a population pharmacokinetic model and the resulting quantiles of concentrations are compared to the observed data. Please refer to Post *et al*.^12^ for more information on VPCs. For the population model, clearance values were assumed to follow a log-normal distribution. The full variance-covariance matrix of random effects was estimated if computationally feasible or otherwise reduced to a diagonal matrix, with *σ*^2^(*η*) representing the estimated population variance of a specific pharmacokinetic parameter. The coefficient of variation (CV%) was calculated via $CV \% =\sqrt{{e}^{{\sigma }^{2}(\eta )}-1}$. Standard errors of parameters were computed via bootstrap statistics with 1,000 samples^13^.

To assess the relationship between iohexol clearance estimates from the population pharmacokinetic model and the Schwartz approach, i.e. for the second step of the evaluation, empirical Bayes estimates (EBE) of clearance values were obtained. Therefore, *η*-shrinkage^14^ of clearance estimates was demanded not to exceed 5%. Distributional assumptions were relaxed by employing a nonparametric algorithm implemented in NONMEM. Additionally, standard errors of EBEs^15^ were assessed using bootstrap results. For the third step, the BIS equations were subsequently re-estimated based on the obtained three-compartment iohexol clearance EBEs, and a leave-one-out cross-validation was used to assess bias, mean squared and mean absolute error. Models were fitted to N-1 subjects, i.e. leaving out a single subject, and predictions were assessed on the clearance EBE of the left-out subject. For the purpose of comparison, this step was also carried out using Schwartz iohexol clearance estimates for the fitting step and validating the resulting model based on the three-compartment clearance estimates.

If a three-compartment model performed better than a two-compartment in the previous steps, an additional simulation evaluation was carried out. The aim of this evaluation was to assess the influence of a third compartment on Schwartz estimates. Therefore, iohexol concentrations were simulated using the developed three-compartment population pharmacokinetic model (570 virtual subjects, sampling times 10, 20, 30, 60, 120, 240 and 300 minutes, iohexol bolus of 3235 mg) and the Schwartz approach was applied to the resulting concentration-time profiles. The goodness of fit was assessed and obtained iohexol clearance estimates were compared to the simulated clearances.

## Electronic supplementary material

Supplementary Information

## Data Availability

The datasets analyzed during the current study are available from the corresponding author on reasonable request.
