# Supplementary material for: Using a three-compartment model improves the estimation of iohexol clearance to assess glomerular filtration rate
Source: Sci Rep. 2018 Dec 7;8:17723. doi: 10.1038/s41598-018-35989-x (PMC6286374; doi:10.1038/s41598-018-35989-x)
Supplement: Supplementary file 1 — Supplementary Information [file 41598_2018_35989_MOESM1_ESM.docx]

**Using a three-compartment model improves the estimation of iohexol clearance to assess glomerular filtration rate**

Max Taubert^1^*, Natalie Ebert^2^, Peter Martus^3^, Markus van der Giet^4^, Uwe Fuhr^1+^, Elke Schaeffner^2+^

^1^Department I of Pharmacology, University Hospital Cologne, Cologne, Germany

^2^Institute of Public Health, Charité Universitätsmedizin Berlin, Berlin, Germany

^3^Institute for Clinical Epidemiology and Applied Biostatistics, University Hospital Tübingen, Tübingen, Germany

^4^Department of Nephrology, Charité Universitätsmedizin Berlin, Berlin, Germany

^+^Both authors contributed equally

*Corresponding Author:

Max Taubert

Department I of Pharmacology, Center for Pharmacology, Clinical Pharmacology Unit
University Hospital Cologne (AöR)
Gleueler Straße 24
50931 Cologne
Germany
Email: max.taubert@uk-koeln.de
Tel: +49-(0)-221-478-86716 21
Fax: +49-(0)-221-478-7011

**SUPPLEMENT**

| **Parameter estimates of the iohexol base model** |
| --- |
| \| **parameter** \| **median [95% CI]** \| **CV%** \| \| --- \| --- \| --- \| \| CL [L/h] \| 3.44 [3.33-3.55] \| 33 \| \| V1 [L] \| 6.40 [5.81-7.19] \| 40 \| \| Q1 [L/h] \| 3.45 [2.95-3.78] \| 41 \| \| V2 [L] \| 5.43 [5.23-5.67] \| 27 \| \| Q2 [L/h] \| 12.0 [9.14-14.4] \| 51 \| \| V3 [L] \| 3.26 [2.68-3.83] \| 63 \| \| prop. error (*10^-3^) \| 0.468 [0.302-0.660] \| - \| \| add. error [ng/mL] \| 2.22 [0.79-3.98] \| - \| |
| **Supp. Table 1** Parameter estimates for the iohexol base model as obtained from bootstrap statistics with 1000 samples. Medians, 95% confidence intervals (CI) and coefficient of variation (CV) for clearance (CL), central volume of distribution (V1), intercompartmental clearances (Q1, Q2), peripheral volumes of distribution (V2, V3) and the proportional and additive residual error. |
| **Goodness of fit plots of the iohexol base model** |
| 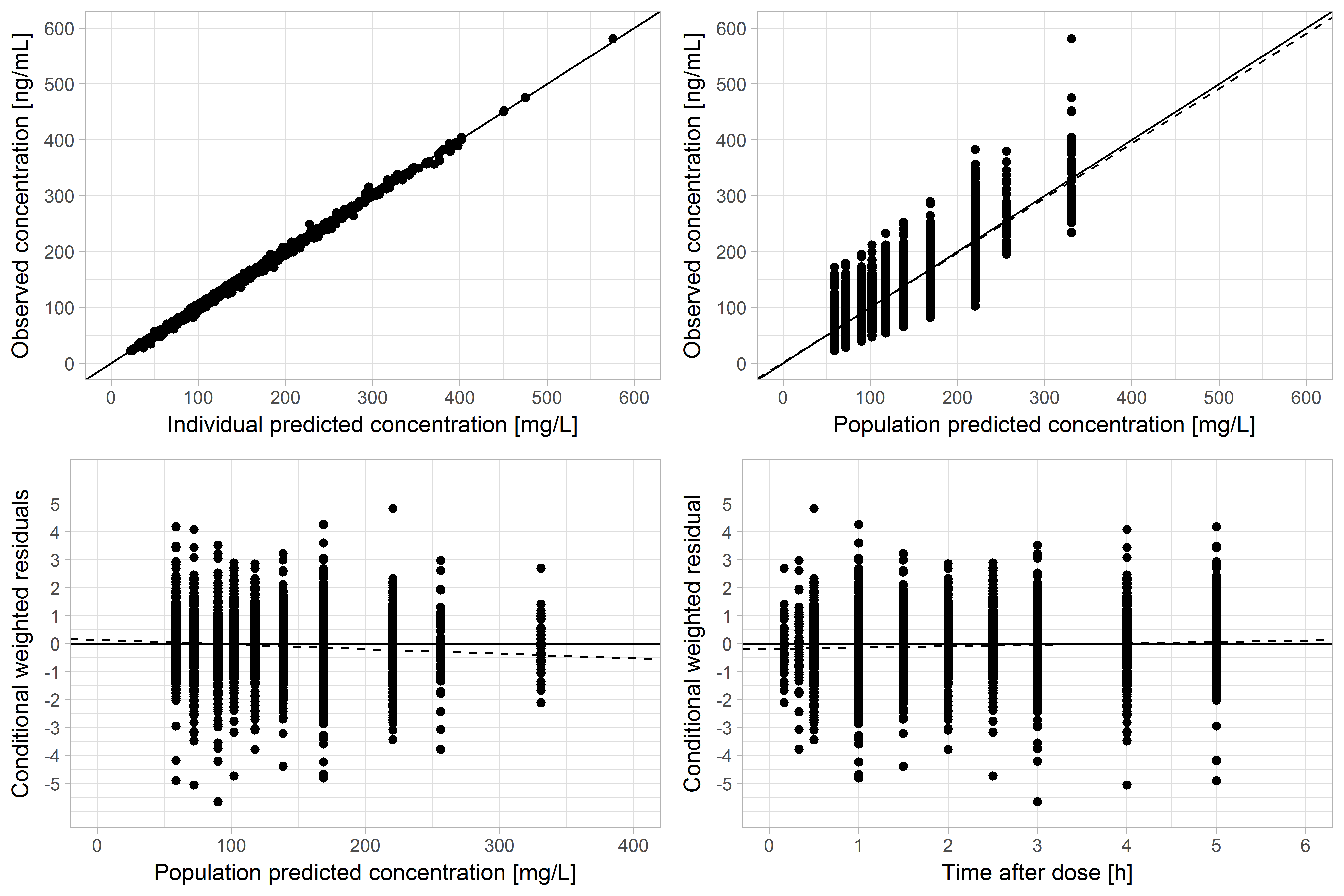 |
| **Supp. Figure 1** Standard goodness of fit plots for the basic population pharmacokinetic model. Observed concentrations are plotted against individual and population predicted concentrations from the model (top row). Conditional weighted residuals are plotted against population predicted concentrations and the time after dose (bottom row). Dashed lines indicate lines of best fit. |
